# The time-varying effects of geopolitical risk on mutual fund risk taking

**DOI:** 10.1371/journal.pone.0303766

**Published:** 2024-06-17

**Authors:** Jie Liu, Zhenshan Chen, Yinglun Zhu, Yangfa Chen, Yaoye Huang

**Affiliations:** 1 College of Economics and Management, Fujian Agriculture and Forestry University, Fuzhou, China; 2 School of International Economics, China Foreign Affairs University, Beijing, China; University of Balamand, LEBANON

## Abstract

Based on a time-varying parameter vector autoregression model with stochastic volatility (TVP-VAR-SV), this paper investigates the dynamic effects of geopolitical risk on mutual fund risk taking in China across three-time horizons and at three selected time points. Overall, the impulse responses are time-varying and we find a negative effect of geopolitical risk on mutual fund risk taking until 2015, with the short-term effect being the most pronounced, suggesting that when professional investors such as mutual fund managers are faced with the stock valuation uncertainty due to a geopolitical shock, they choose to reduce market risk exposures. After 2015, the short-term effect begins to diminish and gradually turns positive, which could be explained by the fact that with the increasing abundance and diversification of investment instruments, fund managers have more effective investment tools and more sophisticated trading strategies to hedge against geopolitical risk, rather than reducing market risk exposure. Further, we explore the heterogeneous effects of eight types of geopolitical risk and three types of mutual fund. The results indicate that the effect of geopolitical actions is stronger than that of geopolitical threats, while the effect of narrow geopolitical risk is stronger than that of broad geopolitical risk. Moreover, we find that the response of the risk taking of growth funds to the geopolitical risk is weaker than that of balanced and income funds.

## 1. Introduction

Frequent geopolitical risk events, such as the 9/11 terrorist attacks, the Iraq war, the Diaoyu Islands conflict between China and Japan, the Paris terror attacks, the North Korean nuclear crisis and the Russia-Ukraine war, have exacerbated global economic uncertainty and have had a profound impact on financial markets. For instance, in the aftermath of the 9/11 terrorist attacks in 2001, we witness a 5.2% drop in the Standard & Poor’s 500 index during the week that followed [1]. Geopolitical risks have raised concerns and attention among regulators and investors. The Bank of England acknowledges that macro-uncertainty shocks, including geopolitical risk and economic policy uncertainty, are expected to create Economic Post-Traumatic Stress Disorder, characterized by heightened caution among households, firms, and financial markets, stemming from the anticipation of future risks and generating a heightened sensitivity to downside tail risks [2]. A survey by Morgan Stanley also suggests that US fund managers will reduce their risk exposures in the face of the increasingly geopolitical risk (Please check https://longportapp.com/en/news/88823085 for more details). With the growing prominence of mutual funds in China’s wealth management industry [3], it has become increasingly crucial to examine the behavior of mutual funds in response to geopolitical risk shocks. Understanding the timeframe and extent to which mutual funds adjust their risk-taking strategies is essential for investors to accurately evaluate the expected returns and risks associated with their investment portfolios to optimize investment decisions, and has significant policy implications for regulators seeking to mitigate excessive market volatility stemming from geopolitical risks and maintain stability in financial markets.

While we acknowledge that previous literature has extensively examined the various aspects of the impact of geopolitical risk on the macroeconomic, asset pricing, and corporate finance [4–16], our research focuses on the effect of geopolitical risk on mutual fund risk taking, which has not been specifically studied. Furthermore, most of the existing literature investigate the cross-sectional determinants of mutual fund risk taking from a micro perspective [17–22]. However, there is a dearth of literature that examines the time-series variation in mutual fund risk taking from a macro perspective. In particular, to the best of our knowledge, there is no literature that focuses on the time-varying impact of geopolitical risk on mutual fund risk taking.

Geopolitical risk, encompassing wars, acts of terrorism, and political tensions between nations, is widely recognized as a macro-level exogenous systemic risk [8]. The inherent nature of geopolitical risk makes it difficult for investors to effectively mitigate its impact through traditional diversification strategies [1]. The literature suggests that risk-averse or ambiguity-averse investors, including fund managers, may reduce their risk taking when faced with increased uncertainty [23–25]. Moreover, [26] further highlight that mutual funds lack efficient investment vehicles compared to hedge funds, and typically lack significant market timing capabilities. Consequently, mutual funds are more inclined to shield themselves against the impact of geopolitical risk and curtail the potential loss of fund returns by lowering their market risk exposure. Based on a TVP-VAR-SV model, this paper investigates the time-varying impact of geopolitical risk on mutual fund risk taking, uncovering its dynamic and nonlinear nature. Additionally, we specifically focus on the time-varying response of mutual fund risk taking after significant geopolitical events, such as the Iraq war, the Diaoyu Islands dispute between China and Japan, and the Russia-Ukraine war. Furthermore, our analysis explores the heterogeneous effects of various types of geopolitical risk on different types of mutual funds.

Our study makes valuable contributions to the literature in the following four respects. First, albeit there have been extensive discussions on the influencing factors of mutual fund risk taking [3,17–22], the literature primarily focus on the micro perspective, such as the education and experience of fund managers, lacking an in-depth understanding of whether and how macro factors can impact mutual fund risk taking. Our study complements the literature by providing original evidence on how geopolitical risks act as an external shock and impact the market risk exposure of mutual fund.

Additionally, we contribute to the growing literature on the economic consequences of geopolitical risk [4–16,27–30], by providing original evidence that the fund risk taking is significantly correlated with geopolitical risk. To the best of our knowledge, this paper is the first specific research exploring the impact of geopolitical risk on funds’ investment styles, particularly risk taking tendencies.

Moreover, our study distinguishes itself from all of the prior research, which employ linear regressions to examine factors that affect fund risk taking [3], with the implicit assumption that the relationship is time-invariant. We instead expand upon the notion that the geopolitical risk has a time-varying and non-linear effect, which has two possible explanations. On the one hand, the geopolitical risk itself has significant time-series variation, with peaks around major geopolitical events such as the 9/11 terrorist attacks and the Iraq war [8]. On the other hand, the impact of geopolitical risk varies over time. For instance, with the occurrence of geopolitical conflict events in recent years, such as the Paris attacks, the US-North Korea nuclear crisis, the US-Iran tensions, and the Russia-Ukraine war, fund managers have not only gained more effective methods of hedging risk, but have also benefited from the availability of diverse investment tools, allowing them to implement more sophisticated strategies in mitigating the impact of geopolitical risk, rather than simply reducing market risk exposure. Therefore, we effectively take into account the time-varying effects of geopolitical risk based on a TVP-VAR-SV model proposed by [31].

Further, we examine the heterogeneity of the impact of different types of geopolitical risk, aligning with the existing literature that highlights significant differences between geopolitical threats and geopolitical actions [1,32]. Overall, our findings not only contribute to a deeper understanding of the effects of geopolitical risk, but also have valuable implications for policy makers, fund investors, and fund managers. For policymakers, it is imperative to recognize the potential for portfolio reallocations of mutual funds and market volatility triggered by geopolitical events. A proactive approach of price stabilization and liquidity support is essential to mitigate the contagious effects of financial risks stemming from geopolitical uncertainties. For fund investors, understanding the nuanced responses of mutual funds to geopolitical risks is crucial in making informed asset allocation decisions. Investors must align their asset allocations with their risk appetites and leverage empirical insights on fund risk-taking behaviors to assess expected returns and risks accurately. For fund managers, the availability of sophisticated investment instruments offers new opportunities to hedge against geopolitical risks. Fund managers must embrace financial innovation, fully utilize these instruments, and master corresponding trading techniques to navigate the increasingly complex market environment caused by geopolitical risks.

The rest of the paper is structured as follows: Section 2 reviews the relevant literature. Section 3 introduces the model and the data used in our analysis. Section 4 presents the empirical results of the TVP-VAR-SV model, and section 5 concludes.

## 2. Literature review

Our study establishes a relationship between geopolitical risk and mutual fund risk taking and is therefore relevant to both parts of the literature. Geopolitical risk refers to the risks associated with wars, terrorist attacks and interstate tensions that disrupt the normal course of international relations [8]. The economic consequences of geopolitical risks have been extensively explored in the literature [4–16]. From a macroeconomic perspective, [8] find that geopolitical risks lead to a contraction in economic activity, which is reflected in lower investment and employment. At the same time, peaks in geopolitical risk are associated with a higher probability of recession, downside risks to GDP and lower expected GDP growth. Moreover, the literature finds that perceptions of geopolitical risk discourage foreign direct investment, while effective governance lessens the impact of geopolitical risk by decreasing policy uncertainty [6].

From an asset pricing perspective, elevated geopolitical risk dampens stock market liquidity [9], devalues exchange rates [10], lowers stock return and bond yields, increases stock market volatility [11,13], and suppresses investor sentiment [16]. Major financial assets, such as stocks, bonds, and commodities, have been exposed notably to geopolitical risk. Numerous studies have primarily focused on the impact of geopolitical risk on stock pricing. For example, [8,30] find that during periods of high geopolitical risk, industries with higher sensitivity to geopolitical risk experience a significant downward pressure in stock returns. Similarly, [13] indicate a significantly negative impact of global geopolitical risk on stock prices in the Chinese rare metals sector after 2012. Regarding the cause of this effect, [29] demonstrate that geopolitical risk reduces household stock market investment primarily due to differences in investors’ risk preferences, rather than the sensitivity to geopolitical risk in specific industries. However, contrary to the findings of [8,11,13,33] argue that global geopolitical risk significantly impact stock return volatility in emerging market rather than returns. On the other hand, [34] focusing on six Gulf Cooperation Council (GCC) countries, find that global geopolitical risk loses predictive power for stock prices when risk-adjusted returns are considered. These studies collectively indicate that geopolitical risk may have heterogeneous effects on stock price performance in different countries and at different times.

As for the impact of geopolitical risk on the bond market, the literature finds a long-term negative correlation between U.S. Treasury bond yields and global geopolitical risk [27]. Additionally, [1] suggest that only exceptionally high levels of geopolitical risk affect bond yields, as it takes prominent news coverage to capture investor attention and alter their investment behavior. [33] also indicate that geopolitical risk impacts both the yield and volatility of the Islamic bond market.

As for the impact of geopolitical risk on the commodity market, [8,28,34] establish that the geopolitical risk demonstrates a robust predictive capability for crude oil volatility, particularly within the Gulf Cooperation Council (GCC) countries. In the context of the Chinese market, the geopolitical risk exerts varying short-term effects on a range of commodities, including composite commodities, energy commodities, agricultural commodities, industrial metals, and precious metals [13]. Moreover, the extent to which different types of commodities are affected by geopolitical shocks varies significantly. The empirical evidence provided by [15] indicate that geopolitical risk exerts the most substantial influence on the price volatility of copper and crude oil, as these commodities are highly reliant on external factors. [35] demonstrate that during the Russia-Ukraine war, some commodities including silver, gold, copper, platinum, aluminum, and sugar, act as net transmitters of volatility, while wheat, oats, and lead exhibit a more pronounced net receiving effect.

From a corporate finance perspective, the literature suggests that geopolitical risk increases uncertainty in the business environment and external financing [4], affects corporate dividend payout policies by raising firms’ cash flow uncertainty and the risk of financial distress [5], reduces foreign direct investment [6], decreases merger and acquisition (M&A) transactions [7], and inhibits corporate investment [8]. Further, the effect of political connection on a company’s exposure to geopolitical risk remains a topic of debate. [4] establish that politically connected firms can mitigate the adverse impact of geopolitical risk on corporate investment, while [5,36] suggest that the effects of geopolitical risk are mainly present in firms whose operations are involved in geopolitical events. While acknowledging that the literature has extensively examined the economic consequences of geopolitical risk, there is a research gap about the impact of geopolitical risk on the investment decision of mutual fund, particularly the risk taking of mutual fund.

In recent years, mutual funds have gained significant prominence in China’s asset management industry, as a growing number of investors have increased their allocation to mutual funds within their portfolios [3]. In order to better understand the risk exposure associated with investing in mutual funds, it is crucial to investigate the factors that drive fund managers’ risk taking behavior. Existing research predominantly focuses on analyzing mutual fund risk taking at the micro level. This line of literature explores various personal characteristics of fund managers and argues that those who have attended prestigious universities, possess higher levels of education, and have more experience tend to exhibit a greater willingness to take on higher levels of risk [17,19]. In contrast, [20] find that fund managers from prestigious institutions, highly educated and experienced in the field prefer lower risk taking. However, [18] note that the level of education or the quality of education is not significantly related to the market risk taken by funds. Instead, they find that fund managers with longer tenure are more inclined to take less systemic risk. In addition to personal characteristics, the literature has also explored the determinants of fund managers’ risk taking behavior from the perspectives of unemployment risks and pay incentives. For example, [21] investigate how the combination of unemployment risks and pay incentives impacts fund managers’ risk taking. Further, [22] find that fund manager ownership can serve as an incentive adjustment mechanism to mitigate excessive risk taking triggered by agency problems.

Compared to the majority of literature that explores the factors influencing fund managers’ risk taking at the micro level, there is a scarcity of research that examines macro-level factors [3]. We fill the research gap by investigating the time-varying impact of geopolitical risk on mutual fund risk taking through a TVP-VAR-SV model.

## 3. Methodology and data

### 3.1 TVP-VAR model with stochastic volatility

Utilizing the TVP-VAR-SV model, we aim to meticulously explore the intricate influence of geopolitical risk on mutual fund risk-taking. This approach is necessitated by the observed temporal variations in the impact of geopolitical risk on the stock market, as outlined in prior research [13]. The TVP-VAR-SV model, originally proposed by [31], possesses a distinct advantage in its capacity to capture nonlinear and time-varying relationships among economic variables. This advantage is achieved by accounting for time variation in both the coefficients and the variance-covariance matrix of the additive innovation, as detailed in [31,37,38]. The model is derived from the basic structural VAR model shown in Eq (1).

$$Ay_{t}=\Gamma_{1}y_{t-1}+\cdots +\Gamma_{s}y_{t-s}+\mu_{t},t=s+1,\ldots ,n$$ (1)

Where *y*_*t*_ defines a *k* × 1 vector of observed variables, *t* is the time, *s* stands for lag times and *A*, *Γ*_1_, …, *Γ*_*s*_ are *k*× *k* matrix of coefficients. The disturbance *μ*_*t*_ is a *k* × 1 structural shock with *μ*_*t*_ ~ *N* (0, ∑∑), where ∑ is shown in Eq (2).

$$\sum =(\begin{array}{cccc} \sigma_{1} & 0 & \ldots & 0\\ 0 & \ldots & \ldots & \ldots \\ \ldots & \ldots & \ldots & 0\\ 0 & \ldots & 0 & \sigma_{k} \end{array})$$ (2)

Where *σ* is the standard deviation of the structural shock. Assuming that structural shocks follow a recursive identification pattern with *A*, taking the form of a lower triangular matrix shown in Eq (3).


$$A=(\begin{array}{cccc} 1 & 0 & \ldots & 0\\ a_{21} & \ldots & \ldots & \ldots \\ \ldots & \ldots & \ldots & 0\\ a_{k1} & \ldots & a_{k,k-1} & 1 \end{array})$$ (3)

As a result, Eq (1) can be derived as Eq (4).

$$y_{t}=B_{1}y_{t-1}+\cdots +B_{s}y_{t-s}+A^{-1}\sum \varepsilon_{t},\varepsilon_{t}∼N(0,I_{k})$$ (4)

Where *B*_*i*_ = *A*^-1^*Γ*_*i*_ (*i* = 1,2, …, *s*). We stack the elements in the rows of *B*_*i*_ to form *β* (*k*^2^ × 1 vector), and let *X*_*t*_ = *I*_*k*_⊗(*y*_*t*-1_′, …, *y*_*t*-*s*_′), where ⊗ denotes the Kronecker product. Then, Eq (4) can be simplified to Eq (5).


$$y_{t}=X_{t}\beta +A^{-1}\sum \varepsilon_{t}$$ (5)

Further, assuming all parameters are time-varying, the model can be expanded to Eq (6), which is the TVP-VAR-SV model used in this paper.

$$y_{t}=X_{t}\beta_{t}+A_{t}^{-1}\sum_{t}\varepsilon_{t}$$ (6)

Where *β*_*t*_, *A*_*t*_ and ∑_*t*_ are all specified to be time-varying.

Following [37], let *ɑ*_*t*_ = (*ɑ*_*21*_, *ɑ*_*31*_, *ɑ*_*32*_, *ɑ*_*41*_, …, *ɑ*_*k*,*k-1*_)′ be a stacked vector of the low-triangular elements in *A*_*t*_ and *h*_*t*_ = (*h*_*1t*_, …, *h*_*kt*_)′ with *h*_*jt*_ = log*σ*^2^_*jt*_, for *j* = 1, …,*k*, *t* = *s*+1, …,*n*. Assuming the parameters in Eq (6) to follow a random walk process, i.e. *β*_*t*+1_ = *β*_*t*_ + *μ*_*βt*_, *ɑ*_*t*+1_ = *ɑ*_*t*_ + *μ*_*ɑt*_, and *h*_*t*+1_ = *h*_*t*_ + *μ*_*ht*_, the normal distribution is derived as shown in Eq (7).


$$\left(\begin{array}{c} \varepsilon_{t}\\ \mu_{\beta_{t}}\\ \mu_{a_{t}}\\ \mu_{h_{t}} \end{array})∼N(0,\begin{array}{cccc} I & 0 & 0 & 0\\ 0 & \sum_{\beta } & 0 & 0\\ 0 & 0 & \sum_{a} & 0\\ 0 & 0 & 0 & \sum_{h} \end{array}\right)$$ (7)

Where *β*_*s*+1_ ∼ *N* (*μ*_*β*0_, ∑_*β*0_), *ɑ*_*s*+1_ ∼ *N* (*μ*_*ɑ*0_, ∑_*ɑ*0_) and *h*_*s*+1_ ∼ *N* (*μ*_*h*0_, ∑_*h*0_).

Following [31], we employ the Bayesian approach with the MCMC algorithm to obtain an accurate and efficient estimation of the TVP-VAR-SV model, since the model is a nonlinear model, and estimating the maximum likelihood function requires extensive computational effort and multiple iterations of filtering. The equal-interval and the time-point impulse response functions are our two primary tools for interpreting the model. Specifically, the equal-interval impulse response measures the dynamic time-varying effect of a unit shock in geopolitical risk in each month on mutual fund risk taking, while the time-point impulse response reflects the dynamic relationship between geopolitical risk and mutual fund risk taking at each point in time.

### 3.2 Data source and variable specification

To examine the time-varying relationship between geopolitical risk and mutual fund risk taking, we begin by merging two databases: (i) the Geopolitical Risk Index Webpage (https://www.matteoiacoviello.com/gpr.htm), which provides monthly geopolitical risk indexes; (ii) the CSMAR database (https://data.csmar.com/), which contains financial and accounting data on mutual funds. The monthly data used in our study cover the period from January 2000 to January 2023.

#### 3.2.1 Risk taking measure in the mutual fund industry

The market risk exposure of fund is the most commonly used risk taking measure in the literature [39–44]. Mutual fund managers usually modify the market risk exposure of their portfolios to achieve various objectives, including hedging against the potential impact of geopolitical risk on fund performance. For instance, they may lower risk exposure by selling stocks with higher market risk exposure, moving to stocks with lower market risk exposure, or increasing cash holdings. Conversely, when there are optimistic expectations about the market, the fund manager may increase the fund risk taking by holding stocks with high market risk exposure or by reducing cash holdings. Therefore, as prescribed by [39], the market risk exposure (*β*_*0*_) of a fund is derived through the regression model (8), which is employed to gauge the risk taking. Following [39], we take the average market beta of actively managed mutual funds (*TotalBeta*) as a proxy for the overall risk taking of mutual funds.

$$r_{t}=\alpha +\beta_{0}r_{m,t}+\beta_{1}r_{m,t-1}+\varepsilon_{t}$$ (8)

where *r*_*t*_ denotes the fund excess return in month *t*, *r*_*m*_ is the excess return on the market portfolio, and the risk-free rate is the monthly rate of the one-year Chinese deposit. The regression incorporates the 1-month lagged excess return on the market portfolio, which effectively mitigates the impacts of asynchronous trading, as proposed by [44].

Further, we employ the fund classification data from the CSMAR database, which categorizes actively managed equity mutual funds into growth funds, balanced funds, and income funds based on their self-declared investment objectives and strategies following [45]. Specifically, growth funds primarily seek capital appreciation by investing in growth-oriented stocks. Balanced funds seek a mix of capital appreciation and income by investing in both growth and income stocks. Income funds focus primarily on generating regular income by investing in dividend-paying stocks. Based on the categorization, we measure the risk taking of the three types of funds using their average market beta, i.e. *GrowthBeta*, *BalanceBeta*, and *IncomeBeta*.

#### 3.2.2 Measure of geopolitical risk

The main independent variable is the Geopolitical Risk Index (*GPR*) developed by [8], which is constructed monthly on the basis of an aggregation of newspaper articles on geopolitical tensions published in 11 leading international newspapers. [8] undertake diverse validation procedures for the index to substantiate the index’s efficiency, comprising a formal audit of 7,000 newspaper articles, correlations with significant historical events linked to warfare, terrorism, or global emergencies, and comparisons with subjective views on geopolitical risk.

There are three justifications for utilizing the global measure of geopolitical risk (*GPR*) developed by [8] to gauge geopolitical risk and explore its impact on the risk taking of mutual funds in China. First of all, in the context of economic globalization, it is noteworthy that China is the world’s second-largest economy and has substantial links with the global economy [15]. The Chinese financial market is therefore affected by shocks stemming from both domestic geopolitical risk and global geopolitical risk, such as 9/11 terrorist attack and the Russia-Ukraine war, which have a significant impact on the Chinese financial market [13]. Compared to a China-specific measure of geopolitical risk, the global measure offers a more comprehensive analysis, encompassing global geopolitical risk information, including that relevant to China. Therefore, with respect to research conducted within the context of the Chinese market, the global measure of geopolitical risk is widely adopted [13,15]. Furthermore, one significant advantage of the Geopolitical Risk Index is its low correlation with other uncertainty indices, such as *EPU* (the Economic Policy Uncertainty Index). The correlation between these two measures is only 0.2 [29], aiding us in separating the impact of geopolitical risk from other types of uncertainty that may endogenously influence the mutual fund risk taking. Moreover, the index has the capacity to capture a diverse blend of terrorist acts, political conflicts and wars, which goes beyond the narrow focus on events solely relating to war or terrorism. Therefore, using the index not only enables us to exceed the constraints of distinct event-based measurement methods but also widens the range of our analysis. Additionally, the detailed subcategories of the global *GPR* index allow us to undertake a thorough investigation into how various origins of geopolitical risk affect the conduct of mutual fund risk taking.

**Fig 1:**
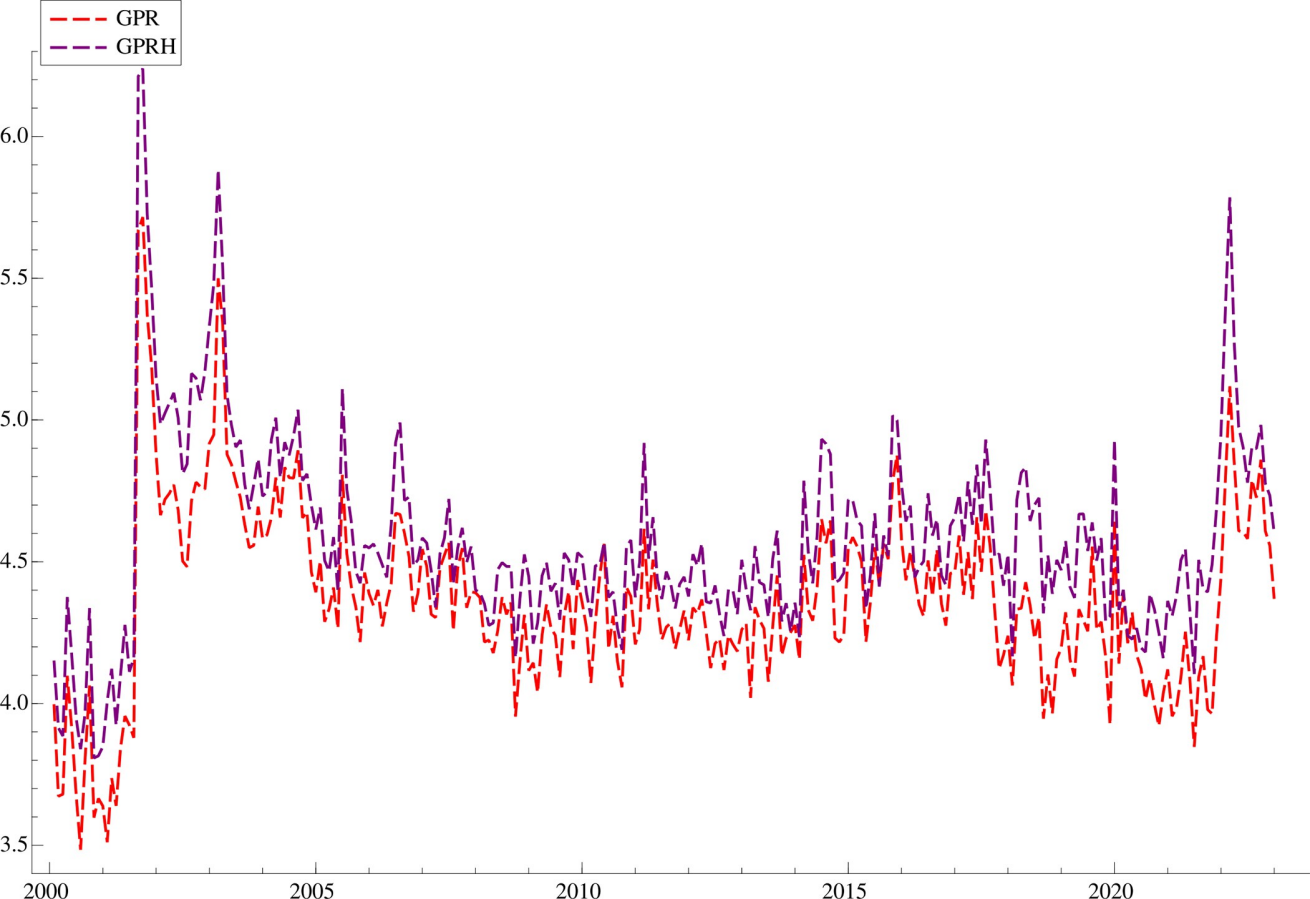
*GPR* and *GPRH* denote the geopolitical risk index and historical geopolitical risk index, respectively.

Additionally, we employ the Historical Geopolitical Risk Index (*GPRH*) developed by [8] as an alternative indicator. *GPR* and *GPRH* exhibit consistent patterns with disparities in two aspects: time scales and data sources. *GPR* encompasses data starting from 1985 and is extracted from prominent news sources such as Boston Globe, Chicago Tribune, Daily Telegraph, Financial Times, Globe and Post, Guardian New York Times, Los Angeles Times, New York Times, Times, Wall Street Journal, and Washington Post. However, *GPRH* entails data spanning back to 1900, and the data is sourced from New York Times, Chicago Tribune, and Washington Post. **Fig 1** illustrates the variation of the geopolitical risk index (*GPR*) and historical geopolitical risk (*GPRH*) from January 2000 to January 2023. The underlying patterns of *GPR* and *GPRH* are consistent, with three distinct peaks observed in 2001, 2003 and 2022. These peaks correspond to significant geopolitical events: the 9–11 terrorist attacks in September 2001, the Iraq war in March 2003, and the Russia-Ukraine war in February 2022. Furthermore, in order to investigate the heterogeneous effects of classified geopolitical risks on the primary data sources. The geopolitical risk index is classified into *GPT*, *GPA*, *GPB* and *GPN*. Specifically, *GPT* and *GPA* capture the geopolitical threats and the geopolitical acts, respectively, while *GPB* and *GPN* capture the geopolitical tensions in the broad and narrow ranges respectively. Similarly, the historical geopolitical risk index is classified into *GPHA*, *GPHT*, *GPHB*, and *GPHN*. Moreover, following [8,14], we logarithmically transform all variables to achieve data stationarity and alleviate heteroscedasticity.

## 4. Empirical results

### 4.1 Unit root tests

Before proceeding to the estimation of a TVP-VAR-SV model, we employ Augmented Dickey-Fuller (ADF) tests and Phillips and Perron (PP) tests to examine the stationarity of the variables after logarithmic transformation. The results are presented in **Table 1**, which indicates that all the variables are stationary in terms of their log levels at the 1% significance level for both ADF and PP tests. Therefore, it makes sense to use the log level form in the model.

**Table 1:** Unit root tests.

Variables	ADF test		PP test	
	Intercept	Trend and Intercept	Intercept	Trend and Intercept
GPR	-5.418***	-5.448***	-5.815***	-5.838***
GPRH	-4.990***	-5.048***	-5.633***	-5.686***
GPT	-5.554***	-5.709***	-6.356***	-6.572***
GPHT	-5.566***	-5.903***	-6.774***	-7.229***
GPA	-4.763***	-5.275***	-5.112***	-5.631***
GPHA	-4.302***	-4.770***	-4.835***	-5.347***
GPB	-5.800***	-6.319***	-5.939***	-6.416***
GPHB	-5.358***	-5.402***	-5.663***	-5.702***
GPN	-4.010***	-5.015***	-5.386***	-5.384***
GPHN	-4.935***	-4.920***	-5.447***	-5.437***
TotalBeta	-7.949***	-10.451 ***	-10.495***	-12.957***
GrowthBeta	-8.784***	-10.203***	-12.419***	-13.877***
BalanceBeta	-9.629***	-10.885***	-11.455***	-12.704***
IncomeBeta	-8.715***	-10.558***	-13.108***	-15.122***

Note: The lag length for the ADF test and PP test are determined based on the Schwarz information criterion (SIC) and the maximum lag is 15. The null hypothesis of the ADF and PP tests is that the variable is nonstationary. Both the model with an intercept and the model with a linear trend and an intercept are employed and the results are generally consistent. ***, ** and * indicate rejection of the null hypothesis at 1%, 5% and 10% significance levels, respectively.

### 4.2 Estimation of selected parameters

To investigate the impact of geopolitical risk on mutual fund risk taking, following [46,47], Markov chain Monte Carlo (MCMC) method based on Bayesian framework is adopted to estimate the TVP-VAR-SV model. The estimation results for selected parameters in the TVP-VAR-SV model are presented in **Table 2**, in which Panels A, B, C, D, E, F, G, and H show the estimation results for the set (*GPR*, *GPRH*, *TotalBeta*), (*GPT*, *GPHT*, *TotalBeta*), (*GPA*, *GPHA*, *TotalBeta*), (*GPB*, *GPHB*, *TotalBeta*), (*GPN*, *GPHN*, *TotalBeta*), (*GPR*, *GPRH*, *GrowthBeta*), (*GPR*, *GPRH*, *BalanceBeta*) and (*GPR*, *GPRH*, *IncomeBeta*), respectively. The findings demonstrates that all of the estimated posterior means are included in the 95% confidence intervals and the standard deviations are small relative to the mean values. Moreover, the Geweke’s diagnostic statistics suggest that all of the parameters cannot reject the null hypothesis at the 5% significance level, indicating that the parameters converge to the posterior distribution. As a result, convergence of the time-varying parameters is successfully achieved, as demonstrated by the diagnostic tests. Further, we observe that the majority of inefficiency factors are below 100 in our study, which are comparable to those reported in [13,14,31]. The largest inefficiency factors are 110.85, which indicates that we get approximately 10000/110.85 ≈ 90 unrelated samples and is enough for effective posterior estimation. Overall, we can safely conclude that the use of MCMC algorithm can effectively estimate the parameters in our TVP-VAR-SV model.

**Table 2:** Estimation results of the main parameters in the TVP-VAR-SV model.

Parameter	Mean	Std	95%L	95%U	Geweke	Inef.
Panel A: Estimates for the set (*GPR*, *GPRH*, *TotalBeta*)						
(∑_*β*_)_1_	0.0225	0.0025	0.0182	0.0279	0.495	15.78
(∑_*β*_)_2_	0.0224	0.0025	0.0182	0.0281	0.005	22.72
(∑_*α*_)_1_	0.0583	0.0144	0.0361	0.0908	0.703	60.75
(∑_*α*_)_2_	0.0648	0.0177	0.0401	0.1080	0.223	78.46
(∑_*h*_)_1_	0.3840	0.0868	0.2364	0.5734	0.080	40.35
(∑_*h*_)_2_	0.3448	0.0875	0.1867	0.5296	0.921	84.34
Panel B: Estimates for the set (*GPT*, *GPHT*, *TotalBeta*)						
(∑_*β*_)_1_	0.0225	0.0025	0.0182	0.0280	0.178	22.61
(∑_*β*_)_2_	0.0222	0.0024	0.0181	0.0275	0.560	14.30
(∑_*α*_)_1_	0.0612	0.0140	0.0381	0.0928	0.004	63.26
(∑_*α*_)_2_	0.0527	0.0117	0.0347	0.0793	0.001	51.46
(∑_*h*_)_1_	0.2763	0.0705	0.1644	0.4376	0.520	78.64
(∑_*h*_)_2_	0.2967	0.0689	0.1669	0.4374	0.548	62.76
Panel C: Estimates for the set (*GPA*, *GPHA*, *TotalBeta*)						
(∑_*β*_)_1_	0.0222	0.0024	0.0182	0.0273	0.517	11.46
(∑_*β*_)_2_	0.0221	0.0024	0.0180	0.0274	0.505	13.49
(∑_*α*_)_1_	0.0551	0.0118	0.0368	0.0822	0.027	50.37
(∑_*α*_)_2_	0.0542	0.0125	0.0356	0.0839	0.920	47.00
(∑_*h*_)_1_	0.4183	0.0969	0.2529	0.6260	0.517	40.07
(∑_*h*_)_2_	0.2944	0.0822	0.1693	0.4891	0.001	90.52
Panel D: Estimates for the set (*GPB*, *GPHB*, *TotalBeta*)						
(∑_*β*_)_1_	0.0222	0.0024	0.0181	0.0276	0.785	12.76
(∑_*β*_)_2_	0.0230	0.0026	0.0186	0.0285	0.305	18.56
(∑_*α*_)_1_	0.0541	0.0123	0.0345	0.0840	0.779	59.90
(∑_*α*_)_2_	0.0670	0.0216	0.0386	0.1160	0.344	84.24
(∑_*h*_)_1_	0.4924	0.0915	0.3258	0.6887	0.554	38.63
(∑_*h*_)_2_	0.2497	0.0678	0.1419	0.4020	0.220	82.28
Panel E: Estimates for the set (*GPN*, *GPHN*, *TotalBeta*)						
(∑_*β*_)_1_	0.0218	0.0023	0.0177	0.0269	0.071	15.01
(∑_*β*_)_2_	0.0224	0.0025	0.0181	0.0277	0.601	15.55
(∑_*α*_)_1_	0.0470	0.0094	0.0323	0.0689	0.138	22.75
(∑_*α*_)_2_	0.0580	0.0150	0.0358	0.0941	0.682	81.91
(∑_*h*_)_1_	0.5482	0.1118	0.3549	0.7901	0.134	52.14
(∑_*h*_)_2_	0.2383	0.0394	0.1735	0.3263	0.032	54.50
Panel F: Estimates for the set (*GPR*, *GPRH*, *IncomeBeta*)						
(∑_*β*_)_1_	0.0224	0.0025	0.0182	0.0279	0.979	10.16
(∑_*β*_)_2_	0.0220	0.0024	0.0181	0.0272	0.951	9.51
(∑_*α*_)_1_	0.0573	0.0151	0.0366	0.0949	0.168	64.44
(∑_*α*_)_2_	0.0693	0.0214	0.0407	0.1264	0.010	87.39
(∑_*h*_)_1_	0.3619	0.0800	0.2197	0.5318	0.213	64.48
(∑_*h*_)_2_	0.3345	0.0887	0.1863	0.5413	0.001	92.05
Panel G: Estimates for the set (*GPR*, *GPRH*, *BalanceBeta*)						
(∑_*β*_)_1_	0.0224	0.0025	0.0181	0.0279	0.060	17.51
(∑_*β*_)_2_	0.0223	0.0023	0.0183	0.0275	0.966	13.48
(∑_*α*_)_1_	0.0600	0.0154	0.0368	0.0979	0.727	54.23
(∑_*α*_)_2_	0.0770	0.0298	0.0416	0.1637	0.075	110.85
(∑_*h*_)_1_	0.2692	0.0797	0.1290	0.4337	0.101	91.53
(∑_*h*_)_2_	0.3307	0.0949	0.1834	0.5592	0.581	92.27
Panel H: Estimates for the set (*GPR*, *GPRH*, *GrowthBeta*)						
(∑_*β*_)_1_	0.0223	0.0025	0.0181	0.0279	0.612	10.78
(∑_*β*_)_2_	0.0219	0.0024	0.0178	0.0270	0.047	7.74
(∑_*α*_)_1_	0.0604	0.0147	0.0382	0.0959	0.457	63.57
(∑_*α*_)_2_	0.0786	0.0326	0.0425	0.1531	0.298	108.75
(∑_*h*_)_1_	0.3990	0.0902	0.2436	0.5948	0.885	68.99
(∑_*h*_)_2_	0.3257	0.0937	0.1677	0.5320	0.636	90.59

Note: This table presents the estimates of selected parameters in the TVP-VAR-SV model by the MCMC algorithm. The results are obtained by generating 10,000 draws from the posterior, and after the initial 1000 samples are discarded. In each panel, we provide the estimates for the TVP-VAR-SV models comprising the geopolitical risks index (*GPR*, *GPT*, *GPA*, *GPB*, and *GPN*), the historical geopolitical risks index (*GPRH*, *GPHA*, *GPHT*, *GPHB*, and *GPHN*), and the risk taking of actively managed mutual funds (*TotalBeta*, *GrowthBeta*, *BalanceBeta*, and *IncomeBeta*). Mean and Stdev denote posterior means and standard deviations, respectively. 95%L and 95%U denote the 95% confidence interval. Geweke denotes the Geweke convergence diagnostics statistics, and Inef denotes the inefficiency factor.

### 4.3 Time-varying effects of geopolitics risk on the risk taking of mutual funds

#### 4.3.1 Time-varying effects at different time horizons

**Fig 2:**
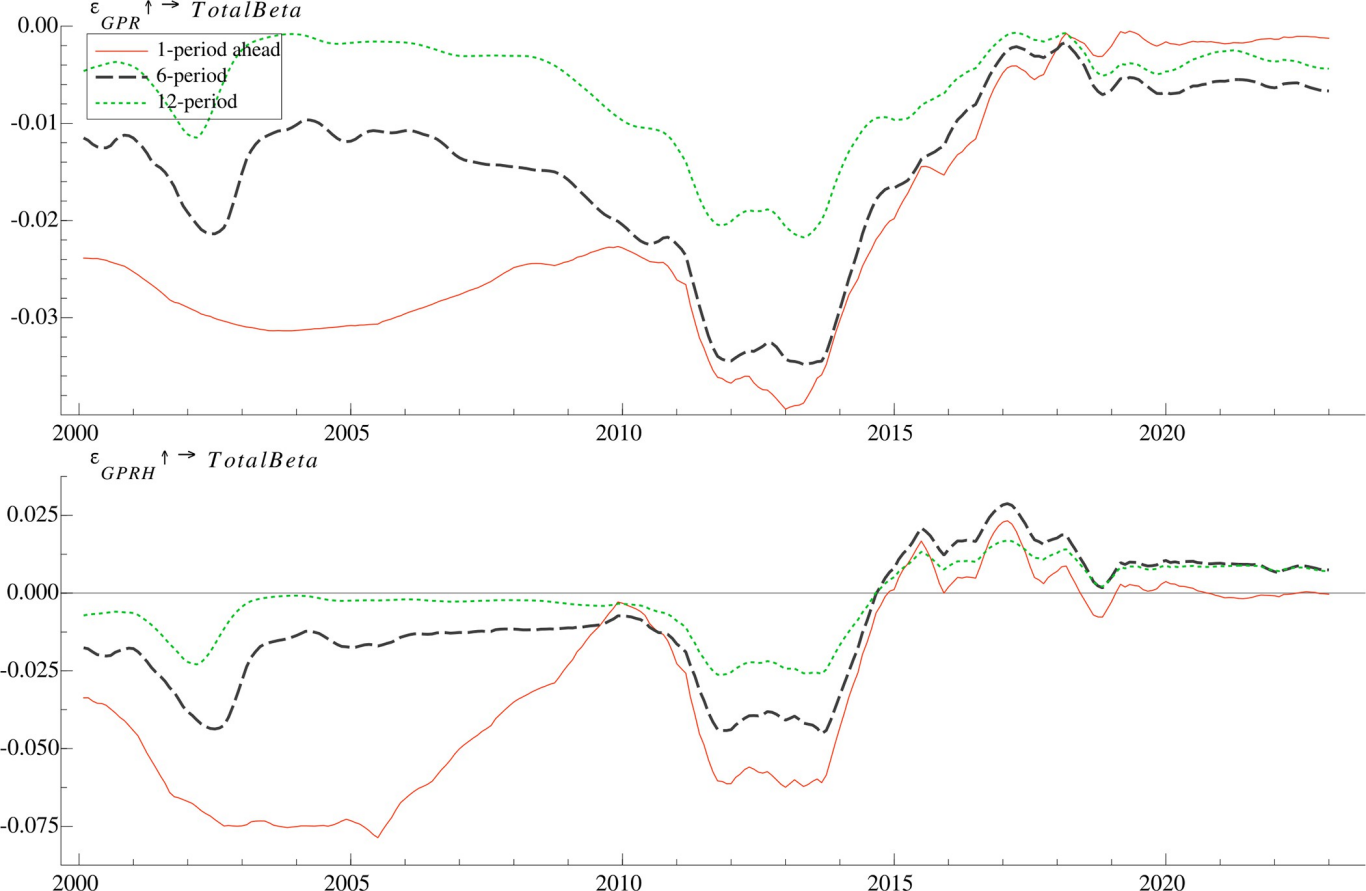
Time-varying impulse responses of mutual fund risk taking to geopolitical risk shock at different lag periods.

We employ the time-varying impulse response function to investigate the dynamic effect of geopolitical risk on mutual fund risk taking, which is measured by the average market beta of actively managed mutual funds. Specifically, we set the time varying impulse response with the cumulated three-dimensional representation, which are 1 period (one month), 6 periods (six months) and 12 periods (one year), representing short-term, medium-term and long-term, respectively. To make the impulse responses on each variable comparable over time, following [14], the amplitude of the shock is set to the time-series average of the stochastic volatility over the sample period. The results presented in **Fig 2** indicate that the responses of geopolitical risk differ significantly over time and the magnitude of the responses varies over different time horizons, justifying the use of the TVP-VAR-SV model.

The responses of mutual fund risk taking (*TotalBeta*) to geopolitical shocks (*GPR*) are found to be significantly negative as expected, indicating that when professional investors such as mutual fund managers are faced with the stock valuation uncertainty due to a geopolitical risk shock, they tend to reduce the market risk loading. Our findings are consistent with the previous literature which claims that unlike their hedge fund counterparts, mutual funds equip less effective investment tools to hedge against macroeconomic uncertainty [26]. Notably, [48,49] point out that geopolitical risks have a significant negative impact on stock prices, which may trigger investors to sell risky assets in search of safer ones until their perception of a stable future is restored [50]. Therefore, in order to hedge against geopolitical risk and prevent geopolitical uncertainty from affecting fund performance, fund managers may choose to hold stocks with lower market risk. In addition, the comparative results across different time horizons indicate that for most of the time in the sample, geopolitical shocks exert the greatest impact on mutual fund risk taking over the next one month and the impact fades over the next six and twelve months, most of which disappears within twelve months. These findings indicate that, overall, the influence of *GPR* on *TotalBeta* concentrates in the short term, which can be explained by the fact that the majority of the effect of geopolitical risks on financial markets is limited to the short term [30,33]. In the face of unexpected circumstances, the fund adopts short-term measures to mitigate its market risk exposure, gradually resuming normal risk taking after the event has been settled [51,52]. Additionally, considering a behavioral finance perspective, geopolitical events only have a temporary influence on investor sentiment and the market panic will unwind gradually [16]. Consequently, following an external geopolitical shock, fund managers tend to react immediately by reducing risk taking to mitigate stress.

Further, the impact of geopolitical shock on mutual fund risk taking is found to be particularly negative in the period from 2012 to 2013, which could be attributed to the ongoing dispute between China and Japan over the Diaoyu Islands and oil exploration rights in the East China Sea. Another typical period in which the risk taking of mutual funds responses significantly to geopolitical risks covering the period from 2003 to 2004, which seem to be associated with Iraq war. However, the response of mutual fund risk taking to geopolitical risk began to weaken after 2014. One possible explanation is that with the increasing abundance and diversification of investment instruments in financial markets, fund managers have more effective investment tools and more sophisticated trading strategies to hedge against geopolitical risk, instead of reducing market risk exposure. Moreover, the time-varying pattern of the response of mutual fund risk taking to *GPRH* is basically consistent with the response to *GPR*, while the response to *GPRH* becomes positive between 2015 and 2018 and alternates between positive and negative after 2018.

#### 4.3.2 Time-varying effects at different time points

**Fig 3:**
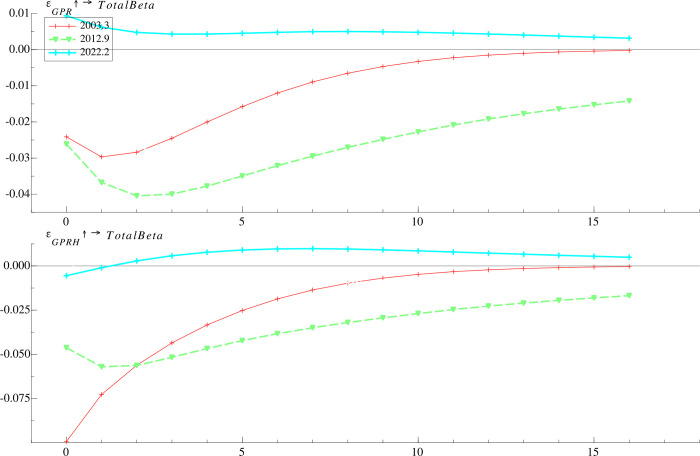
Time-varying impulse responses of mutual fund risk taking to geopolitical risk shock at different time points.

Since the impulse responses of mutual fund risk taking are time-varying, we select three time points to further examine the effects of *GPR* and *GPRH* on the dynamics of *TotalBeta*, which are March 2003, September 2012 and February 2022, corresponding to the Iraq war, the China-Japan Diaoyu Islands dispute and the Russia-Ukraine war, respectively. As shown in **Fig 3**, the negative impact of geopolitical shock reaches its maximum within the fourth month after the geopolitical event, while the magnitude of the responses depends on selected time points. Of the three geopolitical conflicts, the negative response of mutual fund risk taking to the China-Japan Diaoyu Islands dispute is dramatic and persistent. Our findings highlight that when confronted with increasing geopolitical risks related to their home country, fund managers opt for stocks with lower market risk exposure in order to safeguard fund performance from external uncertainties, which is in line with [13,53], suggesting that investors tend to be more responsive to signals from their home country than to international signals. Additionally, we observed a modest but positive response in mutual fund risk taking to the geopolitical risks arising from the Russian-Ukrainian war in 2022, indicating that fund managers might identify investment opportunities amid heightened geopolitical risks and are willing to assume slightly higher risks in pursuit of potential returns. In summary, fund managers adapt their investment strategies flexibly to strike a balance between risk mitigation and capitalizing on opportunities, ultimately aiming to maximize fund performance.

### 4.4 The heterogeneous effects of classified geopolitics risk on the risk taking of mutual fund

#### 4.4.1 Time-varying effects at different time horizons

**Fig 4:**
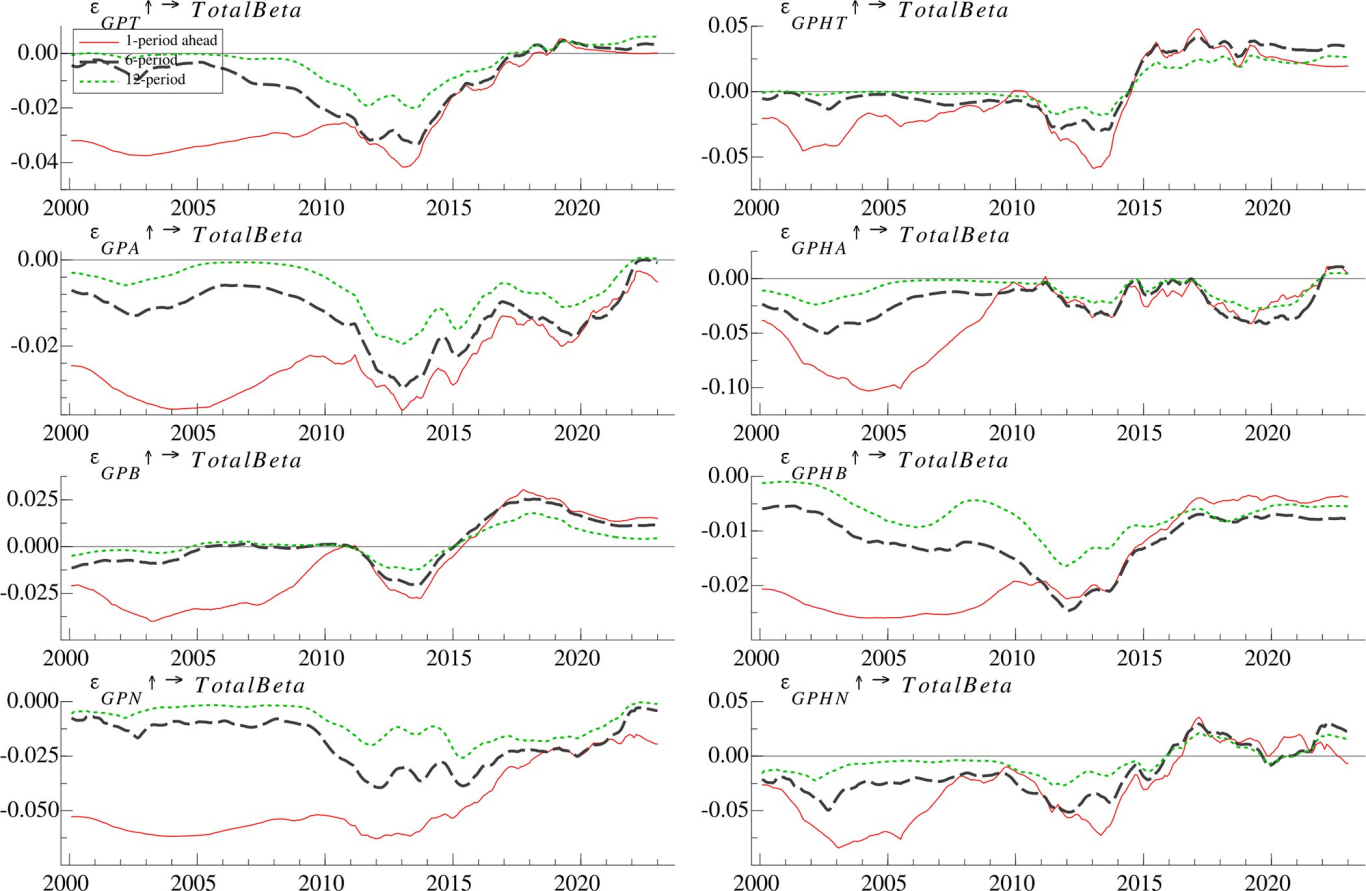
Time-varying impulse responses of mutual fund risk taking to classified geopolitical risk at different time periods.

To investigate the heterogeneous effects of classified geopolitics risks on the risk taking of mutual fund, the geopolitical risk index (*GPR*) is classified into *GPA*, *GPT*, *GPB*, and *GPN*, while the historical geopolitical risk index (*GPRH*) is classified into *GPHA*, *GPHT*, *GPHB*, and *GPHN*. As **Fig 4** shows, the risk taking of mutual fund responds negatively to most types of geopolitical shocks, but the negative effects taper off after 2014, consistent with the pattern of impulse responses to *GPR* or *GPRH*.

The risk taking of mutual fund responds more negatively to *GPHA* than it does to *GPA*, *GPT* and *GPHT* for most of the time, especially during the period from 2002 to 2007, suggesting that the uncertainty generated by geopolitical actions is significantly stronger than that generated by geopolitical threats, forcing fund managers to reduce their market risk exposure more. This phenomenon aligns with the economic rationale that geopolitical actions are more probable to impact financial markets than geopolitical threats [8]. Consequently, geopolitical actions prompt a more significant reaction from mutual funds. Notably, the response to *GPT* turns positive after 2019 and the response to *GPHT* turns positive after 2015, suggesting that mutual fund managers are willing to expose themselves to higher market risk in the presence of geopolitical threats as investment vehicles become more abundant. Mutual fund risk taking responses weaker to *GPB* (*GPHB*) than to *GPN* (*GPHN*), which suggests that mutual funds are more concerned about narrow geopolitical risk than broad geopolitical risk. The responses to *GPB*, *GPHB*, *GPN*, and *GPHN* become less negative or more positive after 2014, suggesting that mutual fund managers are no longer hedging against geopolitical uncertainty by simply reducing their exposure to market risk.

#### 4.4.2 Time-varying effects at different time points

**Fig 5:**
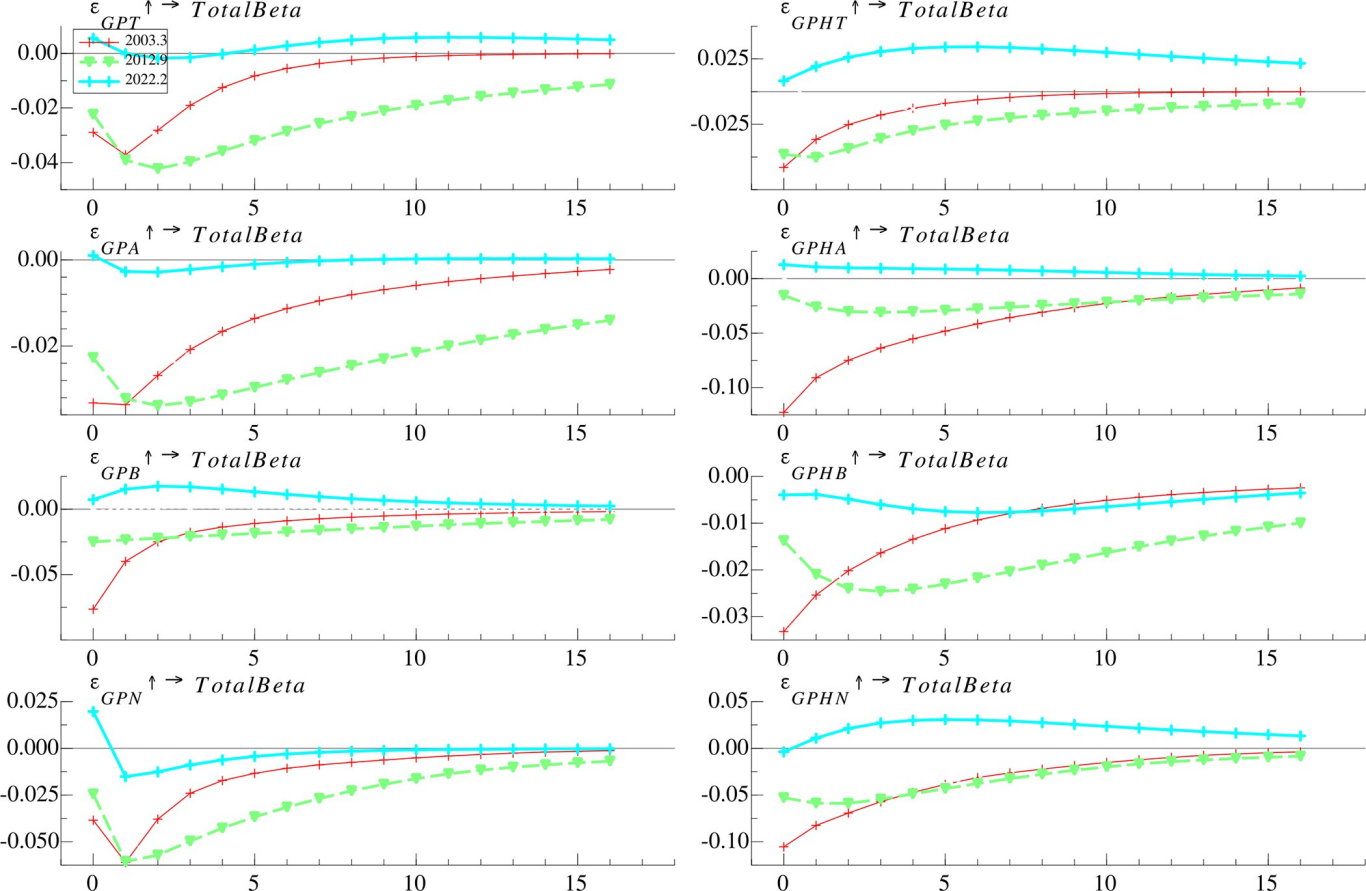
Time-varying impulse responses of mutual fund risk taking to classified geopolitical risk at different time points.

**Fig 5** shows the response of *TotalBeta* to *GPT*, *GPA*, *GPB*, *GPN*, *GPHT*, *GPHA*, *GPHB* and *GPHN* at three selected time points. During the Iraq war, all eight categories of geopolitical risk have an immediate negative impact on *TotalBeta*, and the negative impact would have been maximized within two months. Of these eight categories of geopolitical risk, *TotalBeta* has the most negative response to *GPHA* and *GPHN* in the first month, by approximately 10%. Moreover, during the China-Japan Diaoyu Islands dispute, the response of *TotalBeta* to *GPB* is negative and declines over time, while the response to the other seven categories of geopolitical risk is negative, increases within two months and begins to decay after two months. In addition, *TotalBeta* has a more significant response to *GPN* (*GPHN*) than *GPB* (*GPHB*), suggesting that narrowly defined geopolitical risk is more in line with the mutual fund’s perception of risk. Further, during the Russia-Ukraine war, the responses of *TotalBeta* to different types of geopolitical risk are mixed. Specifically, responses to *GPT*, *GPA*, *GPN*, and *GPHN* are alternately positive and negative over time, responses to *GPHT*, *GPHA*, and *GPB* are positive and diminish after 5 months, and responses to *GPHB* are consistently negative. Our findings suggest that compared to earlier periods, during the Russia-Ukraine war, mutual funds are able to use the effective investment tools to hedge different types of geopolitical risks more accurately, as evidenced by significant differences in the impact of different types of geopolitical risks on the risk taking of funds.

### 4.5 Time-varying effects of geopolitics risk on the risk taking of classified mutual fund

#### 4.5.1 Time-varying effects at different time horizons

**Fig 6:**
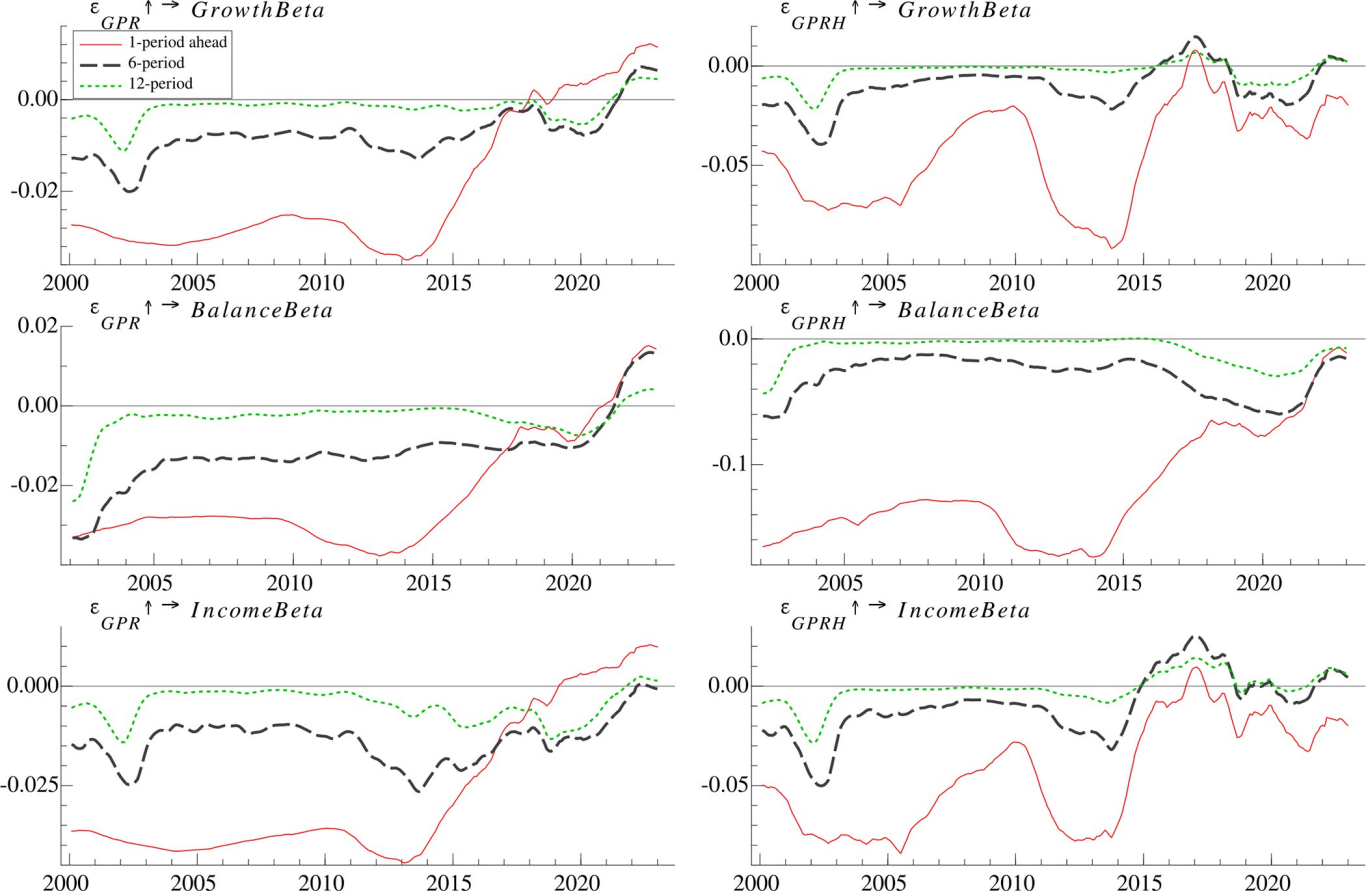
Time-varying impulse responses of risk taking of classified fund to geopolitical risk at different time periods.

Following [45], we categorize actively managed mutual funds into growth funds, balanced funds and income funds, and examine the response of diversified fund to geopolitical risk. As shown in **Fig 6**, prior to 2014, the responses to geopolitical risk shocks exhibit a consistent and significant negative trend, showcasing a higher magnitude in comparison to other time periods. However, following 2014, the negative responses gradually attenuate in magnitude, maintaining a weakening trend that has persisted to the present day. Even for most of the period after 2020, the responses of all three types of funds to *GPR* turn positive. In addition, the risk taking of growth funds is less responsive to geopolitical risk than balanced and income funds, which could be attributed to the fact that growth funds target companies with fast growth rates or high growth potential [45]. As suggested by [45], the high growth is often accompanied by the high risk. As a result, growth funds have a higher risk preference than the other two types of funds, which makes growth funds less sensitive to geopolitical risk.

#### 4.5.2 Time-varying effects at different time points

**Fig 7:**
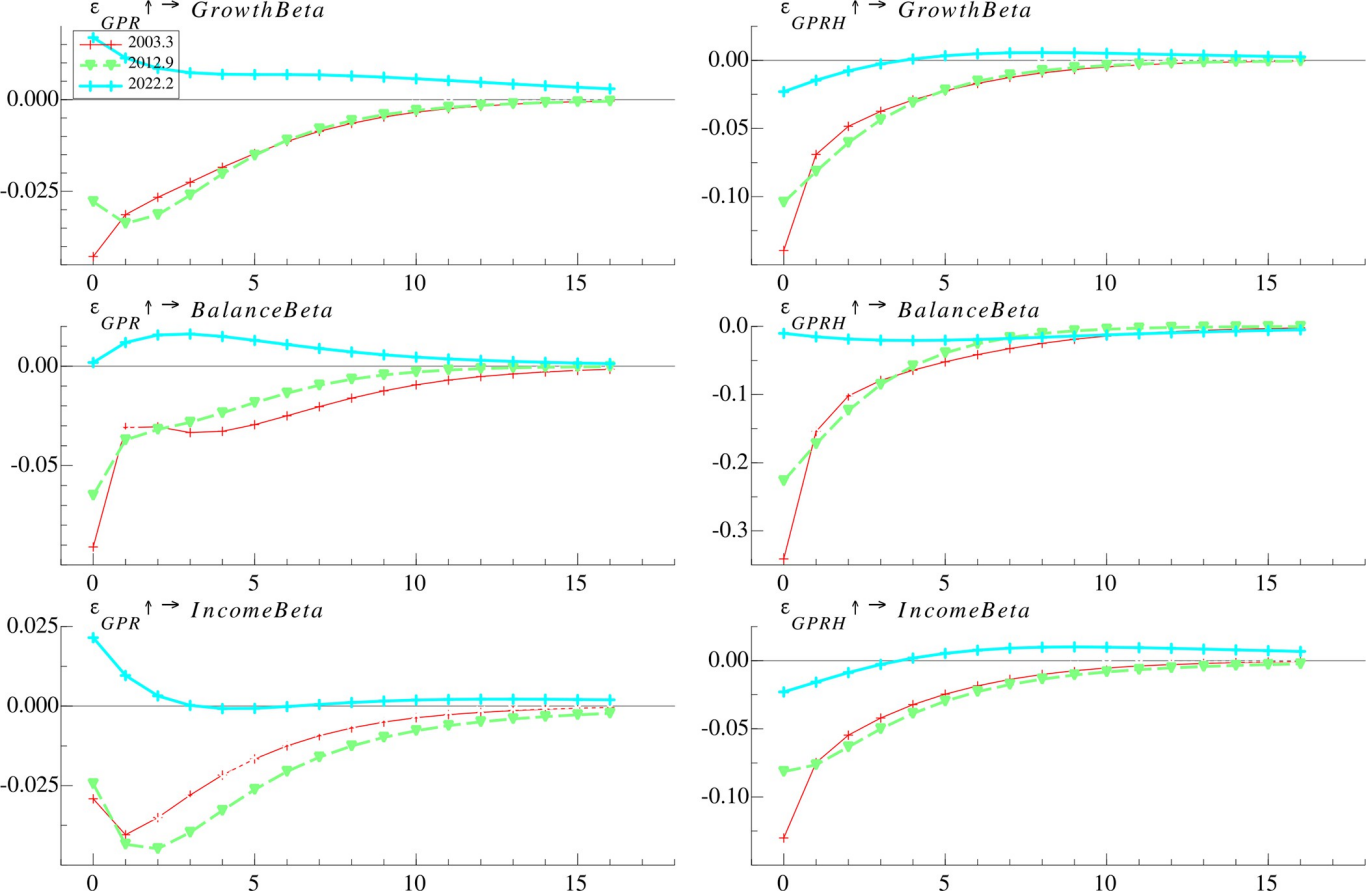
Time-varying impulse responses of risk taking of classified fund to geopolitical risk at different time points.

We further examine the responses of the three types of mutual funds during three selected geopolitical events, i.e. the Iraq war in March 2003, the China-Japan Diaoyu Islands Dispute in September 2012, and the Russia-Ukraine war in February 2022. The results shown in **Fig 7** indicate that during the Iraq war and the China-Japan Diaoyu Islands dispute, the short-term response of the risk taking to *GPR* and *GPRH* is negative for all three types of funds and gradually decays away over time. During the Russia-Ukraine war, the short-term response of the risk taking of growth funds and income funds to *GPR* is positive and gradually recede over time, while the short-term response of balanced funds is almost negligible, peaking within two months before gradually decreasing. On the other hand, the short-term response of growth funds and income funds to *GPRH* is initially negative, but it turns positive after five months. One possible explanation is that these funds raised their cash reserves and diminish risk taking at the outset of the Russian-Ukrainian war due to concerns that the geopolitical risks measured by *GPRH* could impair fund performance. However, following five months, when the fund managers perceived profitable investment prospects as a result of the war [54], they began augmenting their risk exposure to capitalize on potentially gains.

## 5. Conclusions

Frequent geopolitical events have been determined to exert a substantial influence on financial markets [4–16,27–30]. As mutual funds constitute a growing portion of investors’ portfolios [3], their performance and risk exposure are becoming ever more crucial for investors. In this context, based on the TVP-VAR-SV model proposed by [31], this paper employs the geopolitical risk index constructed by [8] to investigate the impact of geopolitical risk on mutual fund risk taking, and to reveal the time-varying and non-linear nature of this relationship. Moreover, we delve into the evolution of mutual fund risk taking in response to specific geopolitical events, such as the Russia-Ukraine war. Additionally, we examine the heterogeneous effects of eight distinct types of geopolitical risks and three different types of mutual funds.

We find a significant negative response of mutual fund risk taking to geopolitical risk prior to 2015, with the short-term effect being the most pronounced. However, after 2015, the short-term effect begins to diminish and gradually turns positive, suggesting that with the increasing availability of investment instruments, mutual funds are more willing to hedge their exposure to geopolitical risk through means other than reducing their market risk exposure. Further, the impact of geopolitical actions is significantly stronger than that of geopolitical threats, prompting mutual funds to reduce their market exposure to a greater extent. Moreover, mutual funds are more concerned about narrow geopolitical risk than broad geopolitical risk. In addition, we find that the response of risk taking of growth funds to geopolitical risk is weaker than that of balanced and income funds, probably because growth funds have a higher risk preference, which makes growth funds less sensitive to geopolitical risk.

Our research makes significant contributions to the literature on mutual fund and geopolitical risk. Albeit there have been extensive discussions on the influencing factors of mutual fund risk taking [3,17–22], the literature primarily focus on the micro perspective, such as the education and experience of fund managers. Our study complements the literature by providing original evidence on how geopolitical risks act as a macro external shock and impact the market risk exposure of mutual fund, contributing to the growing literature on the economic consequences of geopolitical risk [4–16]. Moreover, our study distinguishes itself from all of the prior research [3], which employ linear regressions to examine factors that affect fund risk taking, with the implicit assumption that the relationship is time-invariant. We instead effectively take into account the time-varying and non-linear effects of geopolitical risk based on a TVP-VAR-SV model.

Our study not only fills the research gap by uncovering the time-varying impact of geopolitical risk on fund risk taking, but also provides valuable insights for policymakers, mutual fund investors and fund managers. For policymakers, recognizing the significant effect of global geopolitical risk in shaping short-term mutual fund risk taking is paramount. Geopolitical events can prompt substantial portfolio reallocations by mutual funds, potentially resulting in drastic fluctuations in asset valuations and episodes of insolvency in the market. Consequently, policymakers should consider implementing price stabilization and liquidity support systems to facilitate stable market trading and safeguard market liquidity. Considering the suddenness of geopolitical risks, the proactive approach is essential in guarding against excessive contagion of systemic financial risks that may arise from geopolitical events. For mutual fund investors, allocating assets to funds that correspond with the investor’s risk appetite is a pivotal component of investment decisions while understanding how mutual fund risk taking responds to geopolitical risk allows for a more accurate assessment of the expected return and risk of investing in a fund. Our empirical findings reveal variations in risk taking among growth, balanced, and income funds when responding to geopolitical risks, which can aid fund investors in making more suitable asset allocation decisions depending on the fund type during times of heightened geopolitical risk. For fund managers, the availability of a wider range of investment instruments that offer more options to effectively hedge against geopolitical risk, rather than just reducing exposure to market risk. Fund managers should adopt financial innovation, utilize new financial instruments to their full potential, and master corresponding trading techniques to navigate the increasingly intricate market environment. Furthermore, our findings indicate heterogeneous effects of different types of geopolitical risk. For instance, Chinese mutual funds show more sensitivity to geopolitical risk that involve their own country, such as the China-Japan Diaoyu Islands dispute in 2012, suggesting that regulators and investors should carefully manage different types of geopolitical risks to account for their specific characteristics.
